# Validation of a novel quality of life questionnaire: the Digestion-associated Quality of Life Questionnaire (DQLQ)

**DOI:** 10.1186/s12955-022-01956-4

**Published:** 2022-03-28

**Authors:** Matthew Beke, Alyssa M. Burns, Sydney Weir, Rebecca J. Solch, Taylor C. Judkins, Carmelo Nieves, Bobbi Langkamp-Henken

**Affiliations:** Food Science and Human Nutrition Department, University of Florida, 572 Newell Drive, PO Box 110370, Gainesville, FL 32611-0370 USA

**Keywords:** Digestion, Healthy adults, Validation, Gastrointestinal symptoms, Well-being, Quality of life

## Abstract

**Background:**

Few health-related quality of life (QOL) questionnaires are designed specifically for healthy populations and are specific to gastrointestinal (GI) symptoms even though healthy individuals may frequently experience gas, bloating, constipation, diarrhea, and abdominal pain. The purpose of this study was to develop and validate a tool that could assess the impact of GI symptoms on digestion-associated QOL in otherwise healthy individuals.

**Methods:**

After a review of current literature and with input from experienced GI researchers, a 24-item questionnaire was created. The questionnaire was reduced to 9 items with input from focus groups comprised of healthy adults experiencing GI-related symptoms and through variability analysis. The Digestion-associated QOL Questionnaire (DQLQ) was designed to be sensitive to the physical and mental well-being changes that may occur due to GI symptoms. The DQLQ was assessed for internal consistency reliability (Cronbach’s alpha; McDonald’s omega), test–retest reliability (intraclass correlation coefficient, ICC), and construct validity (Pearson correlations) in a study with healthy, academically stressed, undergraduate students. Convergent validity was evaluated by correlating the DQLQ with gastrointestinal symptom rating scale (GSRS) scores. Divergent validity was assessed by correlating DQLQ scores with stress scores, and bowel satisfaction scores.

**Results:**

A total of 594 students (age 18–30 years) completed the DQLQ. Internal consistency reliability was favorable (n = 594; α = 0.84, ω = 0.84). A high level of agreement and correlation between DQLQ scores was found with the test–retest reliability analysis (n = 273; ICC = 0.89). The questionnaire was shown to have good convergent validity through correlation with the GSRS (n = 594; r = 0.54). Divergent validity was also shown to be appropriate by correlating DQLQ scores with stress (n = 592; r = 0.13, *p* < 0.005), and bowel satisfaction (n = 592; r = 0.18, *p* < 0.001) scores.

**Conclusion:**

The DQLQ is a reliable and valid questionnaire for assessing digestion-associated QOL in healthy individuals.

## Introduction

Clinical research investigating quality of life (QOL) has become increasingly popular in recent years [1]. As clinical laboratory data do not communicate the whole picture related to a condition or an intervention, research is needed to examine the QOL of individuals in addition to the biomedical metrics [2]. QOL questionnaires are frequently used to investigate gastrointestinal (GI) symptoms and their treatments in patients [3–5]. The effect that GI symptoms have on QOL in a healthy population is less established in the literature. Healthy people do experience GI symptoms, as a retrospective cross-sectional study showed that of the more than 52,000 healthy participants, 54% reported experiencing at least one GI symptom within the past seven days [6]. Further, increased severity of GI symptoms has been shown to limit the physical and social functioning and emotional well-being of healthy individuals [7]. Generic QOL tools are available that may be used with healthy individuals [8, 9] but none are specific to digestion-associated QOL.

There are several potential causes for why healthy individuals may experience GI symptoms that impact daily life. For example, GI symptoms or changes in GI function in healthy individuals have been associated with inadequate or more than adequate fiber intake, fermentability of the fiber consumed, or obesity [10–16]. GI symptoms may also worsen with the stress of daily life [17, 18]. Additionally, healthy women typically experience more frequent and severe GI symptoms than healthy men [6, 19, 20] which may be due, at least in part, to menses [21]. These symptoms are likely affecting QOL as North Americans will spend an estimated $43 billion annually by 2022 on dietary supplements, such as prebiotics, probiotics, and functional fibers, to improve GI health and digestion-associated QOL [22]. Unfortunately, little or no data support the efficacy of these interventions. To assess the effectiveness of such interventions in otherwise healthy individuals, as well as to characterize the effect of these GI symptoms on QOL, it is necessary to have a valid, reliable tool. Therefore, the purpose of this study was to develop and validate a tool that could assess the impact of GI symptoms on digestion-associated QOL in otherwise healthy adults. It was also important that this tool be responsive to changes in GI symptoms so that it could be used in a research setting to assess how interventions, such as increasing fiber intake or adding nutritional supplements to the diet, may impact digestion-associated QOL.

## Methods

### Development of the Digestion-associated Quality of Life Questionnaire

**Fig. 1:**
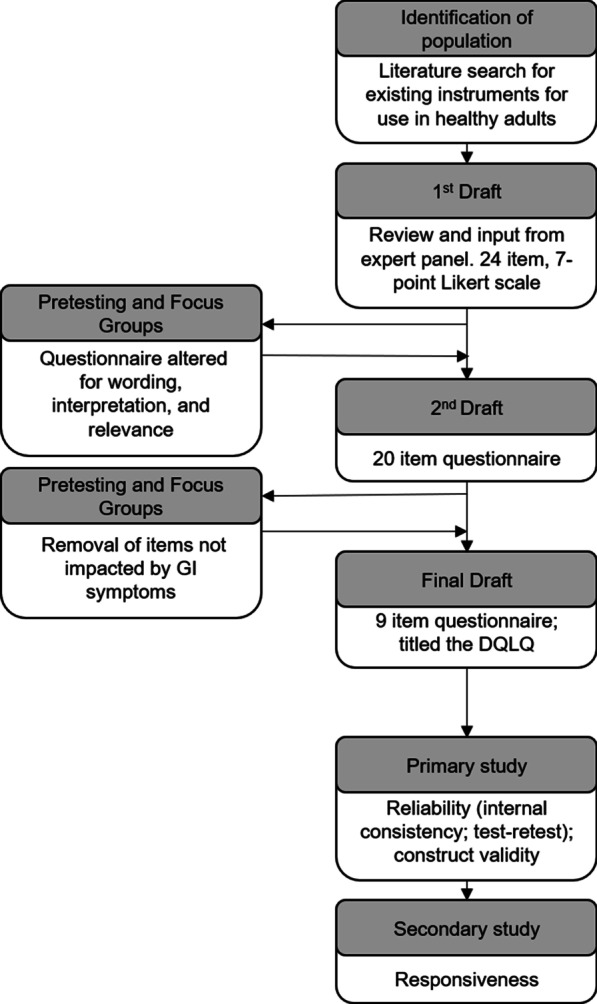
Flow diagram of the development and validation of the Digestion-associated Quality of Life Questionnaire (DQLQ). The first draft of the questionnaire was developed after a review of literature and with input from an expert panel of gastrointestinal researchers. After testing and focus group input, it was revised to a nine-items questionnaire with a seven-point frequency Likert scale. Assessment of the reliability and validity of the DQLQ was then assessed as part of a large clinical trial

In 2015 and 2016, a 24-item questionnaire was generated after a review of QOL questionnaires and GI symptom assessment literature which follows previous developments in GI or QOL research [4, 23–28]. Additionally, input from experienced researchers working in the area of GI health was utilized (Fig. 1). The questionnaire was constructed to capture associations between digestive symptoms and physical activity, dietary intake, worries or concerns, emotional well-being, social interaction, and physical appearance. Three different focus groups of healthy adults (i.e., participants from an initial pilot study and a subgroup of participants from two interventional trials) were used to examine the interpretability and relevance of the items of the questionnaire. The focus group from the initial pilot study included healthy females (n = 12) and males (n = 10) age 22 to 77 years who reported consuming > 25 g of fiber or prebiotic or probiotic supplements daily (unpublished data). It was thought that these participants would be more likely to experience GI symptoms or changes in GI function because of the fiber or dietary supplements. Following administration of the questionnaire, open-ended questions that addressed the wording, interpretation, and relevance of the statements were asked of the focus group.

A revised questionnaire was then prospectively administered as part of two fiber intervention studies in individuals who were consuming a low-fiber diet. It was hypothesized that increasing fiber intake would be associated with more normal, satisfying, and complete bowel movements, as well as GI symptoms. In the first fiber-intervention study, participants (n = 51) received 0, 15, and 25 g of a fermentable fiber (resistant maltodextrin) and GI function was assessed in a crossover study design [29]. As expected, individuals from this study experienced an increase in indigestion-related GI symptoms (rumbling, bloating, burping, and gas) during the periods that the fermentable fiber was consumed. A subset of participants (females: n = 4 and males: n = 2) age 20 to 38 years, volunteered to participate in the focus-group session following the completion of the study (unpublished data).

In a second intervention study, healthy young adults (n = 11 females and n = 9 males) age 18 to 25 years who consumed 15 g of dietary fiber or less (inadequate dietary fiber intake) were asked to consume 39 g of a high fiber (bran) cereal in addition to their baseline intake, making this intervention a significant increase in the amount of dietary fiber consumed [30]. The number of stools per week increased on average by five with the fiber intervention. Data were analyzed to assess the variability in participant responses, and changes in questionnaire scores between intervention and baseline or washout periods. Low variability in responses to an item was interpreted to indicate low relevance (e.g., sleep interruption due to GI symptoms). Focus group participants from both intervention studies were asked similar questions as participants in the initial pilot study focus group.

The Digestion-associated Quality of Life Questionnaire (DQLQ), which assesses digestion-associated QOL over the past week, was then assessed for criterion (postdictive) validity prior to being included in a larger, more comprehensive validation study. Healthy adults (n = 21; 71% female) were asked about the presence (Yes/No) of symptoms covered by the DQLQ each day for seven days, prior to the DQLQ being administered on the eighth day. On the eighth day, the unaltered DQLQ (with frequency answer choices) was administered. Total DQLQ scores were regressed on the total number of “Yes” responses over the previous seven days.

### Validation Study

The DQLQ was prospectively tested in 2017 as an online questionnaire (Qualtrics, Provo, UT) in a randomized, double-blind, placebo controlled clinical trial investigating the effect of probiotic supplements or placebo on stress-associated GI symptoms and immune function during academic stress (i.e., final academic exams) [31]. This study with 594 participants, of which 412 (69%) were female, was deemed appropriate for testing the validity, reliability, and responsiveness of the DQLQ as academic stress and the probiotic interventions had previously been shown to alter GI symptoms, which would likely impact QOL [17, 18]. Potential study participants were recruited via posters, announcements, and electronic mailing lists for this seven-week study. Healthy full-time undergraduate students providing informed consent were included in the study if they were nonsmokers between the ages of 18–30 years (inclusive) who were willing to discontinue consumption of fermented foods or probiotics or immune-enhancing supplements prior to beginning the study. Potential participants were excluded if they were taking any laxatives, did not have access to the Internet during the study period, or were lactating or known to be pregnant.

During the one-week baseline period (i.e., before the start of the interventions) and week 3 of the intervention, participants completed daily questionnaires and the Gastrointestinal Symptom Rating Scale (GSRS) followed by the DQLQ [23]. The GSRS is a 15-item GI symptom severity instrument which assesses five symptom clusters over the past week including reflux, abdominal pain, indigestion, diarrhea, and constipation. A 7-point Likert scale is used to indicate no discomfort (1) up to very severe discomfort (7). Scores for each question were summed for a total score. Daily questionnaires inquired about level of stress, scored 0 (no stress) to 10 (extremely stressed), and bowel satisfaction, scored on a 7-point scale with positive, neutral, and negative response choices. For all study questionnaires, participants could not skip questions, and once they submitted the questionnaires, participants could no longer access the questionnaire to modify their responses.

*Structural validity.* While confirmatory factor analysis (CFA) is typically completed prior to exploratory factor analysis (EFA), CFA was bypassed as the number of factors within the questionnaire could not be identified a priori. Further, a model had not been built using prior research that could be utilized for CFA. Because these principles of CFA were violated, EFA was necessary to explore the underlying structure of the questionnaire. Parallel analysis (Monte Carlo simulation) and a scree plot evaluation were utilized to determine the number of factors to extract (n = 594) [32, 33].

*Reliability*. Internal consistency reliability of the DQLQ was assessed by calculating Cronbach’s alpha (α) and McDonald’s omega (ω) from baseline DQLQ data (n = 594). Coefficients above 0.70 was considered favorable [34].

To examine test–retest reliability, participants were sent an initial DQLQ at 7 AM and a second 10 h after the first. Due to the online format of these questionnaires, it was possible for participants to complete both questionnaires within minutes of each other. For this reason, participants who completed the questionnaires on the day they were sent, in the proper order and four hours or more apart were analyzed. Participants did not have access to their previous responses. Test–retest reliability was assessed in 273 participants by estimating the intraclass correlation coefficient (ICC). The ICC was also assessed for questionnaires that were completed at least eight hours (n = 181) and 12 h (n = 45) apart to analyze responses with potentially less recall bias. An ICC of 0.75 or greater was considered favorable [35].

*Construct validity.* The DQLQ was designed to assess the impact of physical symptoms on digestion-associated QOL. Therefore, Pearson correlation coefficients were determined for DQLQ and GSRS scores using data from the baseline week to support the convergent validity of the DQLQ (n = 594). Altered mental well-being may be related to digestion-associated QOL, even in the absence of physical symptoms (e.g., worry that symptoms may arise). Thus, the DQLQ was compared with stress and bowel satisfaction scores to assess divergent validity as they are different but potentially related constructs to digestion-associated QOL. The average stress scores calculated from daily stress rating during the week of baseline were correlated with DQLQ scores from the same time period (n = 592). The proportion of days during the baseline week that participants were not satisfied with their bowel function was also compared with DQLQ scores. Participants who completed at least one stress and bowel satisfaction questionnaire were included (n = 592). A correlation coefficient of 0.60 or greater was considered strong while a correlation coefficient between 0.30 and 0.60 was considered moderately strong for these convergent validity analyses. Correlations below 0.30 were considered weak and would support divergent validity [36].

*Responsiveness*. Due to unexpected high levels of stress at the beginning of the study when participants were expected to experience low stress, it was not possible to assess the responsiveness of the DQLQ between baseline and week 3 of the intervention when stress-associated digestive events were expected to be more frequent. Thus, the DQLQ was included in another study investigating GI symptoms during the menstrual cycle. Many women report experiencing GI symptoms related to menstruation [37]. Therefore, responsiveness was assessed in regularly menstruating women by comparing DQLQ scores at two points in the menstrual cycle where GI symptoms have been shown to differ [21]. It was expected that DQLQ scores would differ during the different phases of the menstrual cycle. Female participants who menstruated at least monthly were recruited for this five-week observational study. Only women taking oral contraceptive were included to allow for a more homogeneous menstrual pattern, as menstrual cycle lengths may vary. Participants (n = 72) completed the DQLQ on day 7 and day 26 following the start of menstruation (day 1) to assess the difference in digestion-associated QOL during menstrual and non-menstrual weeks, respectively. DQLQ scores from these weeks were compared using the Wilcoxon Signed Rank Test.

*Statistical Analysis.* SPSS version 25 (SPSS Inc, Chicago, IL) was used to conduct all analyses except the Wilcoxon Signed Rank Test (WSRT) for the responsiveness analysis as data were not normally distributed. WSRT was conducting using SigmaPlot (version 14.0; Systat Software Inc, San Jose, CA). WSRT results are expressed as medians (IQR). ICC estimates and their 95% confidence intervals were calculated based on single-rating, absolute agreement, 2-way mixed-effects model.

## Results

### Development of the DQLQ

After feedback from the initial and subsequent focus groups were analyzed, and the variability of responses to the questionnaire was assessed, it was clear that many of the items in the original questionnaire were not impacted by GI symptoms. Thus, the number of items on the questionnaire was reduced to nine and the questionnaire was named the DQLQ. The DQLQ was shown to have favorable criterion validity (r = 0.91; *p* < 0.0001) and can be administered with confidence as scores appropriately reflect events over the past week.

### Validation Study

*Structural validity* Based on the agreement between the parallel analysis and the scree plot, a single factor solution was employed for factor analysis with Maximum Likelihood extraction [38]. All correlations between the items and the latent variable were favorable above 0.30 and ranged from 0.45 to 0.80 [39].

**Fig. 2:**
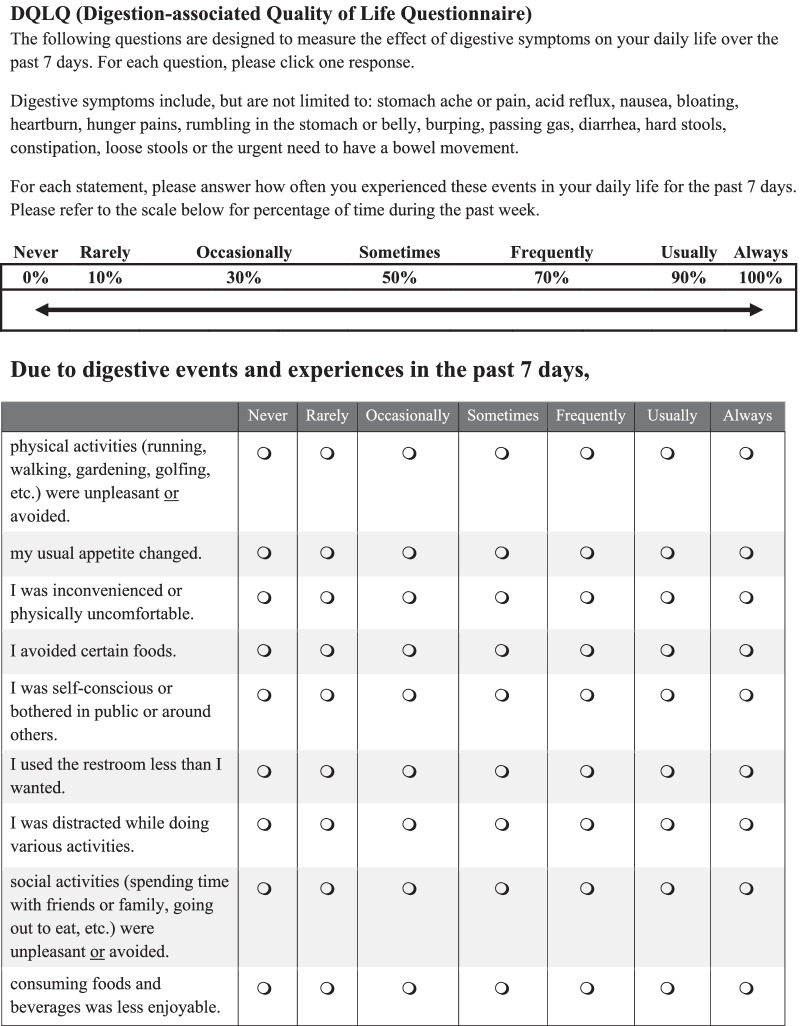
Digestion-associated Quality of Life Questionnaire (DQLQ). Instructions and the questionnaire presented to participants. Administered as an online questionnaire

The DQLQ includes 9 statements that assess how often digestive events and experiences affected the physical and mental aspects of QOL over the past 7 days (Fig. 2). Each statement is scored as follows: never = 0, rarely = 0.1, occasionally = 0.3; sometimes = 0.5, frequently = 0.7, usually = 0.9, and always = 1.0. The total score represents the sum of the responses to the 9 statements with possible scores ranging from 0 to 9. A higher score indicates a lower (worse) digestion-associated QOL.

*Reliability* Internal consistency reliability as measured by Cronbach’s α and McDonald’s ω was 0.84 for both coefficients, suggesting that the variation in items reflects a shared dimension. For the analysis of test–retest reliability, participants completed the questionnaires 9.3 ± 2.7 (mean ± SD) hours apart. The ICC was found to be 0.89 (95% CI: 0.87–0.91) for questionnaires completed at least four hours apart indicating favorable agreement between the two scores and supporting the reliability of the DQLQ. The ICC also indicated a high degree of agreement for questionnaires completed eight hours (ICC = 0.88; 95% CI: 0.84–0.91) and 12 h (ICC = 0.90; 95% CI: 0.83–0.94) or more apart.

*Construct validity*. In the assessment of convergent validity, participant DQLQ data correlated favorably with the GSRS (r = 0.54, *p* < 0.001). The percentage of days that participants were not satisfied with their bowel function weakly correlated with DQLQ scores (r = 0.18, *p* < 0.001). Correlations between the DQLQ and average weekly stress scores were also weak (r = 0.13, *p* < 0.005). These correlations with bowel satisfaction and stress indicate good discriminate validity of the DQLQ.

*Responsiveness*. Median (IQR) DQLQ scores were 1.2 (0.40–2.6) during menstruation when GI symptoms were significantly higher and 0.70 (0.20–1.7; *p* < 0.05) during non-menstrual weeks when GI symptoms were minimal.

## Discussion

Improving the QOL of patients with GI diseases has become a priority for clinicians [3, 4, 40, 41]. However, a significant proportion of healthy adults also experience GI symptoms [6, 42] as evidenced by the billions of dollars being spent annually on dietary supplements to improve digestive health [22]. Consequently, there is a need for a digestion-associated QOL instrument that could be used in research settings to assess the effectiveness of these supplements in healthy adults [22].

The primary aim of this study was to develop and validate a questionnaire to measure the impact that GI symptoms have on digestion-associated QOL in healthy adults. Initial statements for the DQLQ were formulated from the literature and experienced researchers working in the area of GI health. The tool was subsequently tested and refined with input from healthy individuals whose GI function had been altered by increasing daily fiber intake.

During refinement of the questionnaire, special consideration was given to the scoring scale of the DQLQ. Each of the nine items of the DQLQ is scored on a scale of 0 to 1. Thus, total scores can range from 0 to 9. A larger scale (e.g., each item scored from 0 to 10 for a total maximum score equal to 90) may result in large numeric changes that may or may not be clinically relevant. The scale of 0 to 1 was chosen to avoid interpreting clinically insignificant changes in total scores as meaningful.

Test–retest reliability was examined in participants who completed the DQLQ twice on the same day, at least four hours apart. Although there is no standard for time in which a ‘retest’ should be administered in relation to the initial test, enough time after the original test must elapse so that the responder honestly answers the questions again (i.e., considers their present health state rather than their previous responses). Data from questionnaires completed eight or more hours apart, as well as 12 or more hours apart were also examined to ensure test–retest reliability beyond the 4-h retest. Responses were still highly correlated. Additionally, the period between the test and retest cannot be too long such that the tests assess different digestive events or time periods. In the current study, restricting analysis to responses submitted on the same day ensured that the same seven-day period was assessed by participants.

Convergent validity was evaluated by comparing DQLQ scores to GSRS scores. It was predicted that with an increase in GI symptoms (higher GSRS score) that DQLQ scores would also increase, indicating a lower digestion-associated QOL. Divergent validity was examined by correlating average weekly stress scores and bowel satisfaction scores with DQLQ scores. Stress and bowel satisfaction are different but possibly related constructs to digestion-associated QOL. The weaker correlation with stress scores may be explained by the bi-directional communication along the gut-brain axis (i.e., psychological stress can cause GI symptoms and GI symptoms can increase the level of stress). Increased psychological stress is also associated with extraintestinal physical symptoms such as tachycardia, pain, and sleep disturbances [43–45]. Similarly, GI symptoms may predict bowel satisfaction, but bowel satisfaction may not predict digestion-associated QOL.

Responsiveness is the ability of an instrument to detect a relevant change [46]. Menses was chosen as an appropriate life experience to challenge the responsiveness of the DQLQ, as many healthy women report experiencing GI symptoms related to menstruation. Common symptoms experienced during menstruation include abdominal pain, diarrhea, constipation, nausea, and vomiting [37]. Other studies have indicated that a significant portion of female participants experience GI symptoms on premenstrual or menstrual days due to fluctuations in sex hormones [47, 48]. In this study, women were asked to report GI symptoms related to menstruation during menstrual and non-menstrual weeks. Participants also completed the DQLQ at these times [21]. A difference of 0.5 was found between the median DQLQ score during the menstrual phase and non-menstrual phase (1.2 menstrual versus 0.70 non-menstrual). This difference in score reflects the natural physiologic contrast in GI function that healthy adult females normally experience.

Several limitations should be noted. When analyzing test–retest reliability, only participants who responded to both tests on the same day were analyzed. This choice was made to ensure that the same 7-day period was assessed by the participants but may have also introduced recall bias. Additionally, the physiologic causes of GI symptoms leveraged in these studies were not homogeneous. However, this supports the use of the DQLQ in a wide variety of research settings. Finally, the minimal important change or difference in DQLQ score was not investigated. Future research will elaborate further on the smallest change that participants perceive to be important.

## Conclusion

In summary, the DQLQ is a valid questionnaire for the assessment of digestion-associated QOL in healthy adults. Scores correlate with clinically relevant changes in GI symptoms. These findings contribute to the growing research focus on QOL related to digestion and GI symptoms in adults.

## Data Availability

The datasets generated and/or analyzed during the current study are not publicly available but are available from the corresponding author on reasonable request and approval from the University of Florida Institutional Review Board.
